# Conventional and Unconventional Approaches for Innovative Drug Treatments in COVID-19: Looking Outside of Plato’s Cave

**DOI:** 10.3390/ijms22137208

**Published:** 2021-07-05

**Authors:** George J. Kontoghiorghes, Annita Kolnagou, Stella Fetta, Christina N. Kontoghiorghe

**Affiliations:** Postgraduate Research Institute of Science, Technology, Environment and Medicine, 3 Ammochostou Street, Limassol 3021, Cyprus; an.kolnagou@gmail.com (A.K.); stellafet@yahoo.gr (S.F.); c_kontoghiorghe@hotmail.com (C.N.K.)

Thousands of drugs and nutraceuticals along with their combinations can be used to select candidate therapeutics for targeting the transmission, proliferation and the fatal or severe symptoms of the severe acute respiratory syndrome coronavirus 2 (SARS-CoV-2) in order to reduce the unacceptably high mortality rate observed in the coronavirus disease 2019 (COVID-19) pandemic and its associated negative effects on daily life worldwide. About 180 million people have been infected with SARS-CoV-2 and 3.9 million have died since December 2019, the time of the announcement of the first COVID-19 cases. At the same time it is estimated that 11.6 million people are active carriers of SARS-CoV-2, with 99.3% recovering without experiencing any life threatening side effects [1].

Emergency measures have been implemented worldwide for the prevention of COVID-19 transmission and protection against the toxic effects of SARS-CoV-2, with vaccinations promoted as the main shield for reducing the high mortality rate. However, the lack of effective therapies, the absence of global health policies and worldwide co-ordination and the continuous emergence of more toxic variants of SARS-CoV-2 (e.g., gamma and delta), as well as other fallouts such as economic depression, commercial and political differences between nations etc., are likely to drag out the pandemic and the high mortality rate over the next few years. The need for effective drug therapies is still as pressing as it was at the beginning of the pandemic.

A multi-level comprehensive strategy against the COVID-19 pandemic should be considered based on drug targeting methods against all aspects of the viral infection phases, including the mechanisms and related biochemical pathways involved in the fatal and other serious symptoms following infection. Effective drug intervention for any target at any level that can reduce the mortality rate of COVID-19 patients to at least a level similar to that resulting from the influenza virus may result in the end of the need for emergency measures for the COVID-19 pandemic [2]. 

Additional strategies could include the combination of selected drugs with available vaccines in the era of emerging new, more toxic variants of SARS-CoV-2, the planning and implementation of essential health and drug policies based on one world/one health models and other related drug therapeutic approaches. 

The process for new drug selection against COVID-19 could be based on the identification of all specific available targets including targets related to the prevention of transmission, targets involved in the different stages of the life cycle of SARS-CoV-2 or targets related to any of the life threatening symptoms of the infection. Additional targets should be considered for different categories of patients with co-morbidities such as elderly, obese, cardiovascular, pulmonary, renal, diabetic, immuno-compromised and other patients, who are more susceptible to developing serious illness as a result of the SARS-CoV-2 infection [3]. 

The strategy for drug selection could be based on different methods as previously shown in other drug development cases, including the identification of one drug for one target, or of a multi-potent drug for many targets and drug combinations for one or more targets [4,5,6,7,8]. Furthermore, the investigations for drug selection could be based amongst others on personalized medicine parameters including absorption, distribution, metabolism, elimination, toxicity (ADMET) characteristics, drug interactions especially for drugs taken concurrently by patients with co-morbidities, which may have positive or negative effects against SARS-CoV-2 (COVID-19), and also drug interactions with vaccines [3,4,5,6,7,8].

Special consideration should be given to the fatal and other serious symptoms in COVID-19 patients. In particular, SARS-CoV-2 appears to affect mainly the respiratory system but also other systems such as the cardiovascular, gastrointestinal, nervous, immune, and hematopoietic systems [3]. The major symptoms and cause of mortality in COVID-19 patients are associated with a respiratory syndrome and lung damage developed as a result of hyper-inflammation, which is characterized by different stages of progressive deterioration of the respiratory system and of regression from fever to hypotension, coagulopathy, respiratory failure, hypoxia and death [3,9]. In particular, elderly COVID-19 patients with pulmonary and other co-morbidities are more susceptible to the toxic effects of the SARS-CoV-2 infection [3]. 

So far, several drugs with different modes of action have been shown to reduce the rate of mortality and are routinely used in the treatment of different categories and stages of seriously ill COVID-19 hypoxic patients. These include the widely used immunosuppressive drug dexamethasone, the antiviral drug remdesivir, which was initially used against hepatitis C virus and the monoclonal antibody tocilizumab, which is an interleukin-6 (IL-6) receptor antagonist approved for the treatment of inflammation in refractory rheumatoid arthritis patients [3,9,10]. 

The current risk/benefit assessment of different available treatments one year following the COVID-19 pandemic supports the emergency testing and approval of more drugs in addition to vaccines since the current rate of mortality of COVID-19 patients worldwide is unacceptably high and also for many other reasons such as restrictions in traveling, decline in economic activity and general reduction in quality of human life globally. The method of drug selection is similar to many orphan drug development efforts and especially for diseases where there is no available therapeutics [11]. However, in the case of the COVID-19 pandemic the situation is even more urgent because of the large number of fatalities observed [1,11].

The major target areas for drug development could involve all major aspects and stages of COVID-19. In this context, methods and drug formulations could be developed for inhibiting the transmission of the virus among people. For example, drugs could be used for reducing the viral load in the exhalation droplets of carriers of SARS-CoV-2, while at the same time reducing the viral load intake in non-infected individuals. Another area of drug targeting could involve several steps related to viral growth and proliferation including anti-SARS-CoV-2 drugs, as well as inhibition of viral activity involving host cells including attachment, growth and proliferation and viral exit from the host cells. A different major area of drug targeting strategy involves the fatal and other serious toxic side effects of COVID-19 including all stages leading to hyper-inflammation, and also inhibition of all stages leading to endothelial damage, the vicious circle of oxidative stress toxicity, the acute respiratory distress syndrome (ARDS), hypoxia and multi-organ and systemic failure [3,12]. 

A different drug targeting strategy may involve pathways associated with abnormal biochemical, haematological and other changes recorded during the different stages of COVID-19, and especially changes associated with fatalities such as increases in IL-6, C-reactive protein, serum ferritin, D-dimer etc., most of which the etiology has not yet been fully evaluated. 

Additional large numbers of drug targeting options could involve specific proteins, protein receptors, mRNA, specific biochemical pathways and cells playing a key role in the proliferation of SARS-CoV-2, as well as the outcome of the COVID-19 infection [13,14,15]. Similarly, many more drug targeting strategies could involve the modulation of activity of proteomic, genomic, transcription and other factors associated with the mRNA and proteins of SARS-CoV-2 and affected host cells.

Many more drug targeting strategies using conventional and unconventional approaches could be envisaged for the control of the COVID-19 pandemic, which could involve all other stages and sub-stages of the disease including drugs for the control of hypoxia, treatment of sepsis etc. all of which play a significant role in the increased mortality rate observed in COVID-19.

The high mortality rate observed in the COVID-19 pandemic, demands the emergency testing and approval of drugs and nutraceuticals which are widely used in other diseases. In particular, the therapeutic window for the use of such drugs appears to range from a few days to a few weeks. In this context, the risk/benefit assessment for this short therapeutic time window favors rapid approval and administration of sufficiently high doses of drugs at the maximum approved range in COVID-19 patients. Despite that many drugs have already been tested in COVID-19 patients, the efforts for new approvals is not very clear, with regulatory authorities worldwide having different approaches. Western countries appear mostly to promote very expensive therapies with monoclonal antibodies, for which the availability to developing countries and also long term toxicity is uncertain. In the meantime, many approved drugs with hundreds of patient years’ of use and safety behind them have not yet received similar attention for testing and approval. The uncertainty surrounding the testing and approval of investigational new drugs (IND) is even higher despite the emergency [11]. 

Insufficient worldwide health policies which includes ineffective prevention measures, inadequate and slow drug screening procedures for emergency medicines, financial inequality among countries etc., can all impede the rate of control over the COVID-19 pandemic and also any efforts for reducing the high rate of mortality. 

New approaches on prevention, improved worldwide vaccination policies and also the rapid introduction of more approved drugs with different targeting potentials could reduce the current unacceptable high rates of mortality in COVID-19 patients. It is proposed that multi-target drug strategies could improve the therapeutic potential against COVID-19 and restore quality of life for people worldwide.

## Data Availability

Not applicable.
